# Supplementary material for: Clinical grade ACE2 as a universal agent to block SARS‐CoV‐2 variants
Source: EMBO Mol Med. 2022 Jul 4;14(8):e15230. doi: 10.15252/emmm.202115230 (PMC9350269; doi:10.15252/emmm.202115230)
Supplement: Supplementary file 1 — Appendix [file EMMM-14-e15230-s001.pdf]

Table of Contents – Appendix

Appendix Figure S1 - Binding of SARS-CoV-2 RBD variants to ACE2

Figure Legend Appendix Figure S1

Appendix Figure S2 - Binding of SARS-CoV-2 Spike variants to ACE2

Figure Legend Appendix Figure S2

Appendix Figure S3 - SARS-CoV-2 neutralization studies

Figure Legend Appendix Figure S3

Appendix Figure S4 - Neutralization of the SARS-CoV-2 Omicron variant by recombinant murine ACE2 (rmACE2) or recombinant human ACE2 (APN01)

Figure Legend Appendix Figure S4

Appendix Figure S5 - Neutralization of the reference strain of SARS-CoV-2 or SARS-CoV-2 Omicron by vaccinees' sera

Figure Legend Appendix Figure S5

Appendix Table S1 - List of mutations of Variants of Concern

Appendix Table S2 - List of primers

Appendix Table S3 - List of antibodies

Appendix Table S4 – p-values for Figures and Appendix Figures

Appendix Figure S1

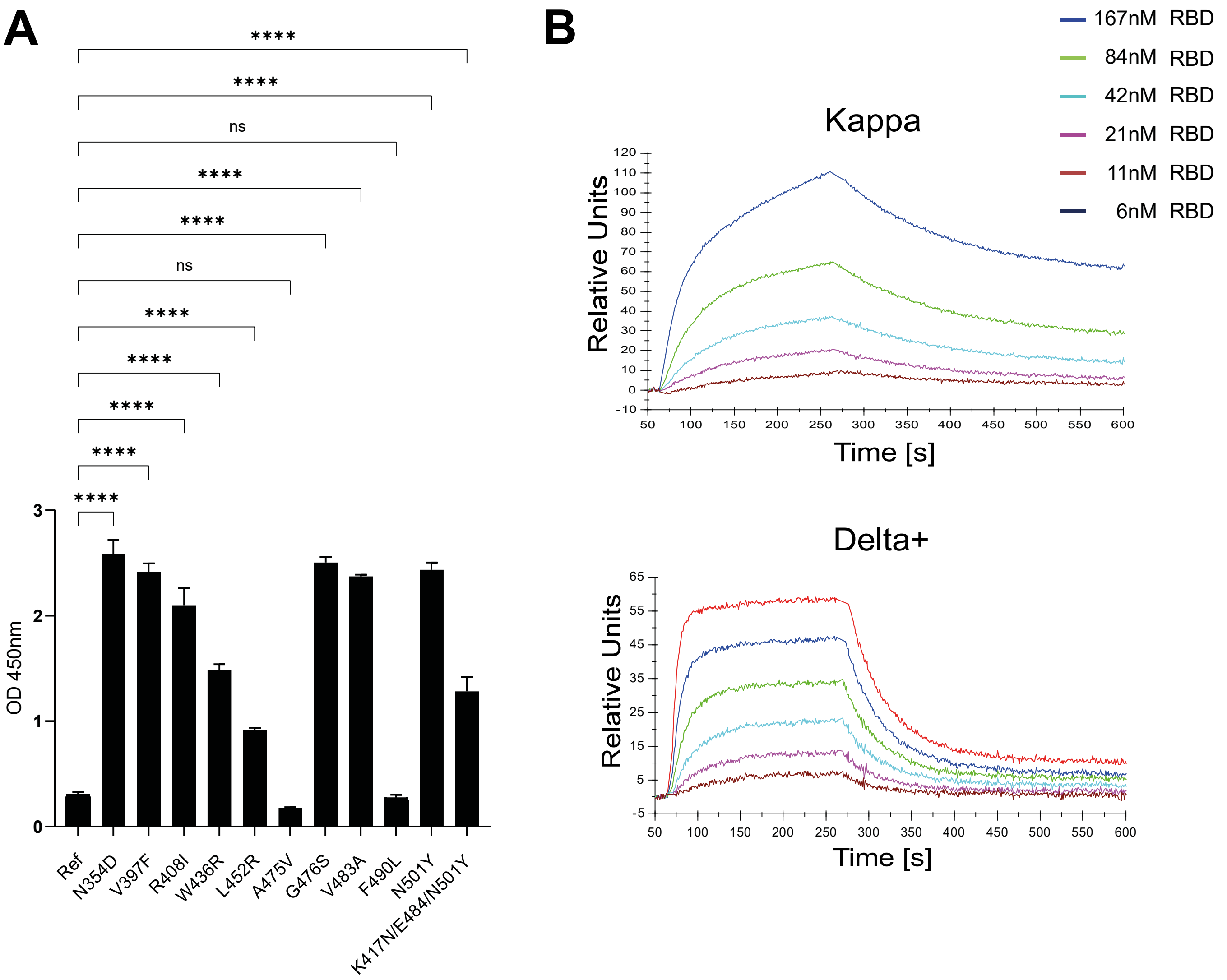

Appendix Figure S1. Binding of SARS-CoV-2 RBD variants to ACE2.

(a) ELISA analysis showing the APN01 binding strength of SARS-CoV-2 RBDs carrying the indicated mutations. x-axis labels indicate the SARS-CoV-2 RBD variant substitutions tested. One representative experiment out of 3 independent experiments is shown. Shown are means of triplicate analyses with standard deviations. Statistical significance is indicated by asterisks (p-value < 0.05: *; p-value < 0.001: ***; p-value < 0.0001: **** as calculated with one-way ANOVA).

(b) Representative SPR sensorgram images for the SARS-CoV-2 RBD/APN01 interaction of the SARS-CoV-2 variant Kappa and Delta+ (K417N, L452R, T478K; see Figure 1b for respective values).

Appendix Figure S2

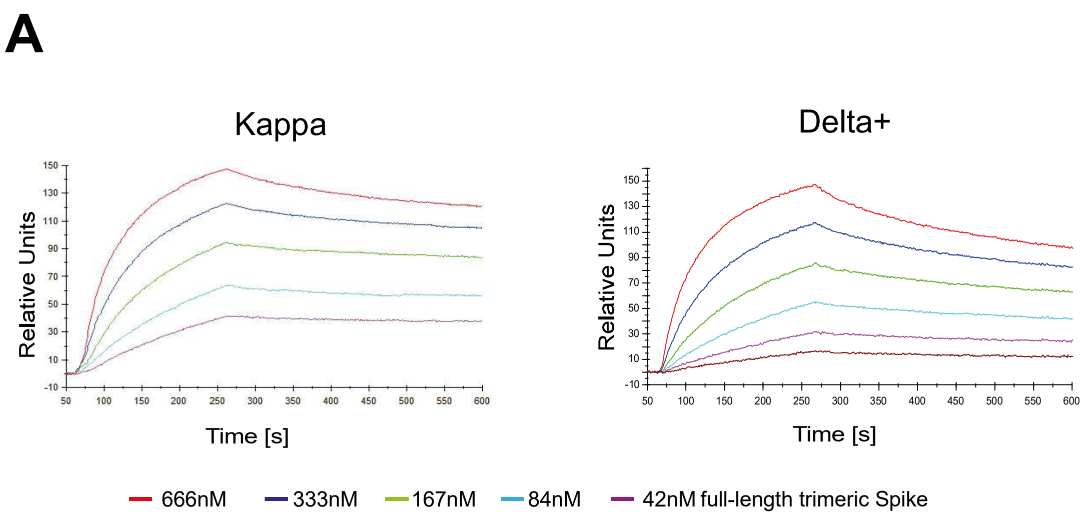

Appendix Figure S2. Binding of SARS-CoV-2 Spike variants to ACE2.

(a) Representative sensorgram images for the SPR analysis conducted with full-length trimeric Spike proteins in the pre-fusion state with APN01. Depicted are SARS-CoV-2 variants Delta+ and Kappa. See Figure 1 **(b)** for respective mutations and Figure 1 (d) for k_a, k_d, as well as K_D values for the interaction of APN01 and the full-length trimeric Spike proteins of the Delta+ and Kappa variants.

Appendix Figure S3

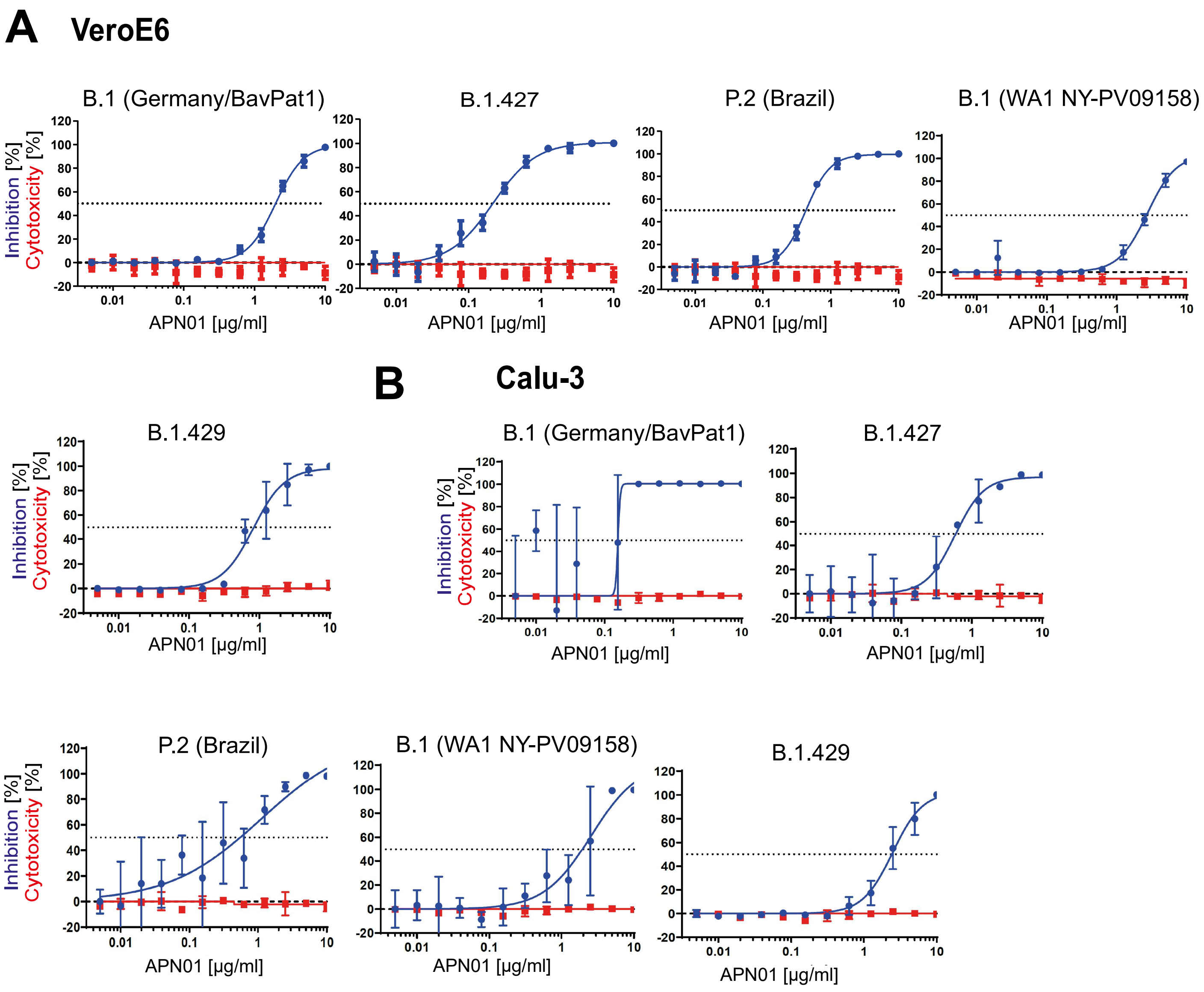

Appendix Figure S3. SARS-CoV-2 neutralization studies.

(a, b) Neutralization of the indicated SARS-CoV-2 isolates (blue line) as well as cytotoxicity of APN01 (red line) in VeroE6 cells **(a)** and Calu-3 cells **(b)**. Analysis was done in quadruplicate with mean and standard deviations shown. Y-axis depicts the percentage of neutralization and cytotoxicity, respectively. See Figure 3 (a) for associated information on the strains and infection conditions and Figure 3 (c) for the IC_{50} and IC_{90} values.

Appendix Figure S4

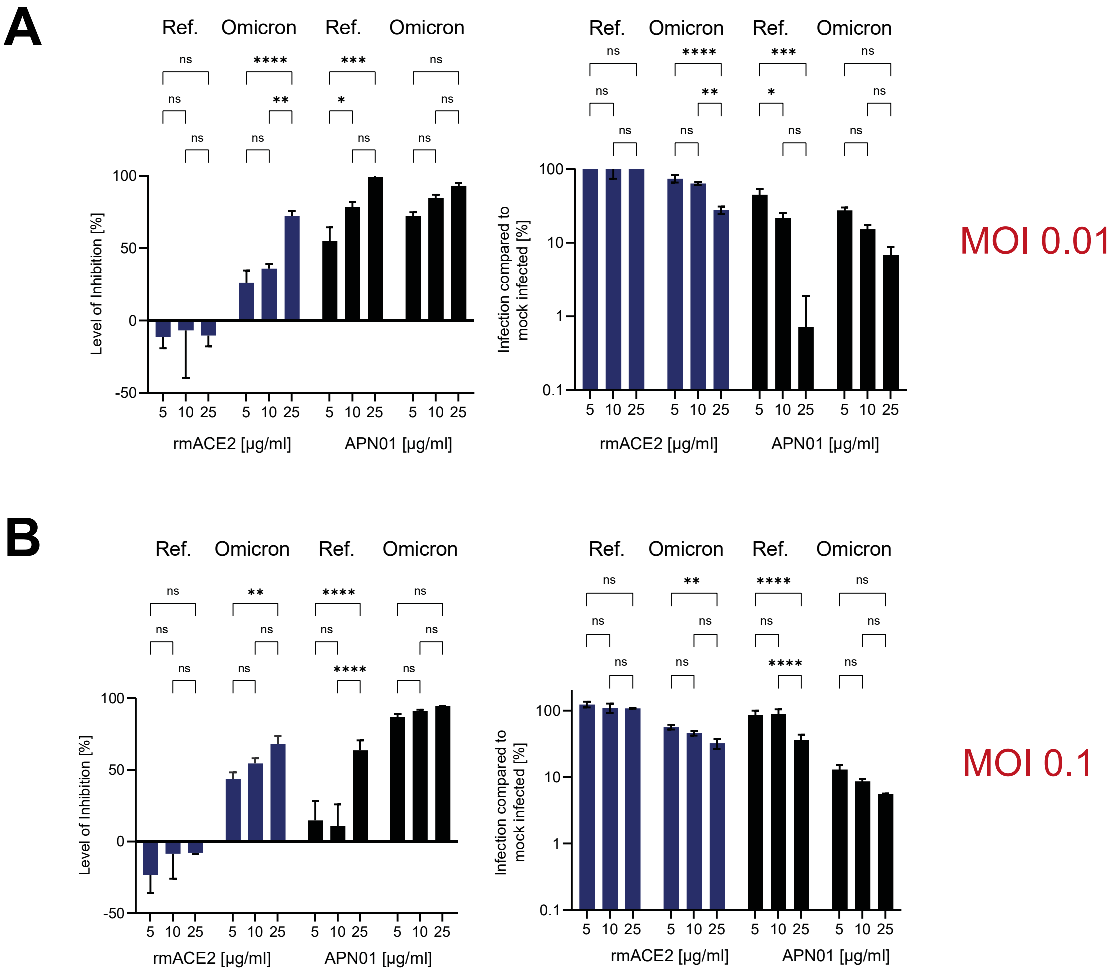

Appendix Figure S4. Neutralization of the SARS-CoV-2 Omicron variant by recombinant murine ACE2 (rmACE2) or recombinant human ACE2 (APN01).

(a, b) Calu-3 cells were infected with SARS-CoV-2 Omicron with a MOI of 0.01 **(a)** or MOI of 0.1 **(b)** after pre-incubation with the indicated doses of either rmACE2 (purple bars) or APN01 (black bars). Left panels in **(a, b)** depict the level of inhibition, right panels in **(a, b)** depict percent infection compared to mock infected controls. Shown are means of triplicate analyses with standard deviations. Statistical significance is indicated by asterisks (p-value < 0.05: *; p-value < 0.01: **; p-value < 0.001: *** as calculated with one-way ANOVA.

Appendix Figure S5

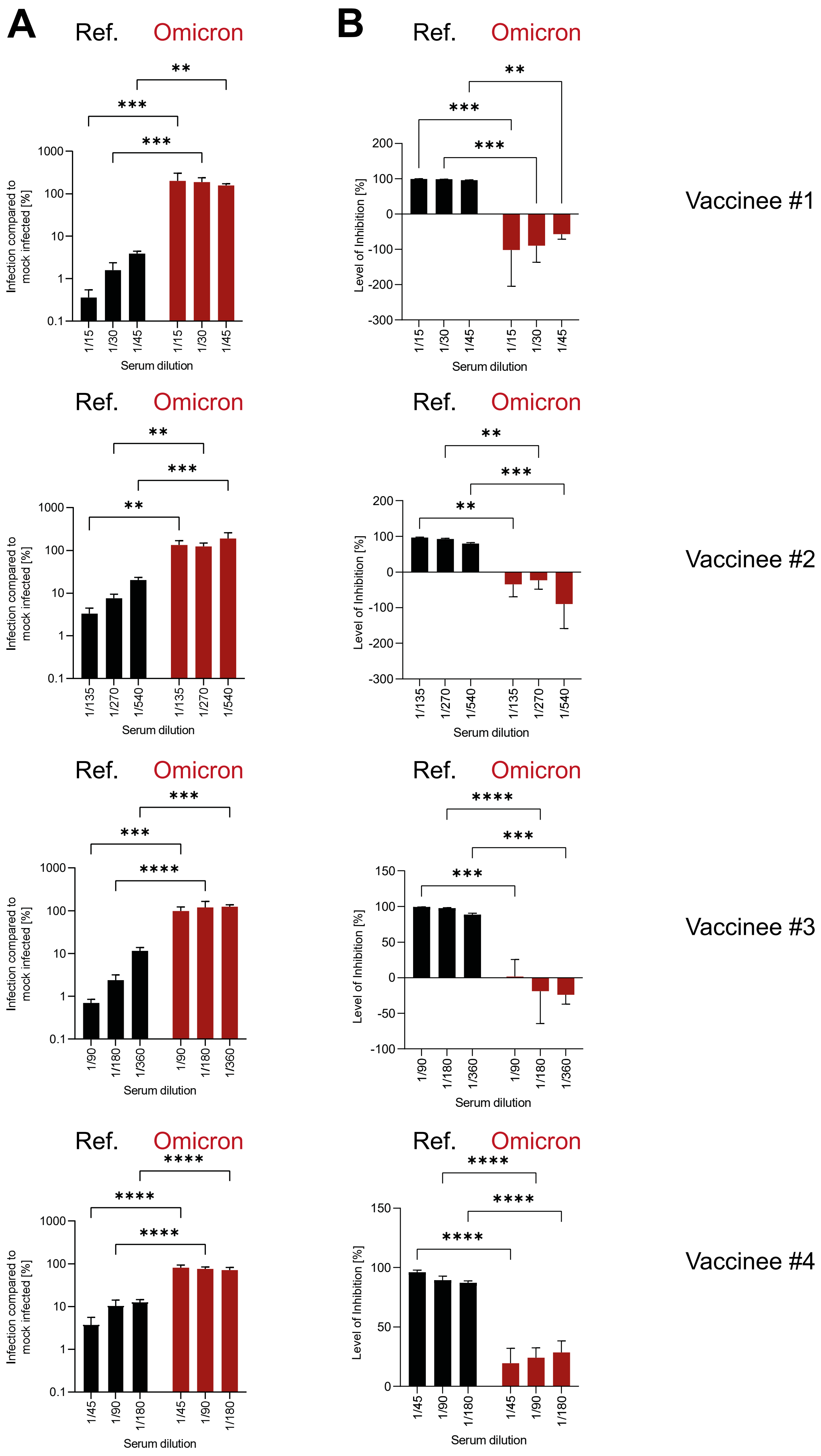

Appendix Figure S5. Neutralization of the reference strain of SARS-CoV-2 or SARS-CoV-2 Omicron by vaccinees' sera.

(a, b) VeroE6 cells were infected with either the SARS-CoV-2 reference strain (black bars) or Omicron (red bars) after pre-incubation with the indicated dilutions of vaccinee sera. Sera were taken 5-7 weeks after the second vaccination with the mRNA vaccine *Comirnaty* (BNT162b2; Biontech/Pfizer). Graphs depict the infection as compared to mock infected cells with the indicated dilutions of vaccinee sera **(a)** or the level of inhibition of infection **(b)**. Data are shown as mean values of triplicate analyses with standard deviations. Statistical significance is indicated by asterisks p-value < 0.05: *; p-value < 0.01: **; p-value < 0.001: *** as calculated with two-way ANOVA.

Appendix Table S1 – List of mutations of Variants of Concern

Position	Wuhan	Alpha	Type	Gene
241	C	T	SNP	ORF1ab
913	C	T	SNP	ORF1ab
3037	C	T	SNP	ORF1ab
3267	C	T	SNP	ORF1ab
3743	C	T	SNP	ORF1ab
5388	C	A	SNP	ORF1ab
5986	C	T	SNP	ORF1ab
6954	T	C	SNP	ORF1ab
11287	GTCTGGTTTT	G	DEL	ORF1ab
14408	C	T	SNP	ORF1ab
14676	C	T	SNP	ORF1ab
15279	C	T	SNP	ORF1ab
16176	T	C	SNP	ORF1ab
**21764**	**ATACATG**	**A**	**DEL**	**S**
**21990**	**TTTA**	**T**	**DEL**	**S**
**23063**	**A**	**T**	**SNP**	**S**
**23271**	**C**	**A**	**SNP**	**S**
**23403**	**A**	**G**	**SNP**	**S**
**23604**	**C**	**A**	**SNP**	**S**
**23709**	**C**	**T**	**SNP**	**S**
**24506**	**T**	**G**	**SNP**	**S**
**24914**	**G**	**C**	**SNP**	**S**
27972	C	T	SNP	ORF8
28048	G	T	SNP	ORF8
28111	A	G	SNP	ORF8
28280	GAT	CTA	MNP	N
28881	GGG	AAC	MNP	N
28977	C	T	SNP	N

Position	Wuhan	Beta	Type	Gene
241	C	T	SNP	ORF1ab
1059	C	T	SNP	ORF1ab
3037	C	T	SNP	ORF1ab
5230	G	T	SNP	ORF1ab
9438	C	T	SNP	ORF1ab
9968	G	T	SNP	ORF1ab
10323	A	G	SNP	ORF1ab
11288	TCTGGTTTT	-	DEL	ORF1ab
12747	C	T	SNP	ORF1ab
14408	C	T	SNP	ORF1ab
17999	C	T	SNP	ORF1ab
18657	C	T	SNP	ORF1ab
21191	C	T	SNP	ORF1ab
**22206**	**A**	**G**	**SNP**	**S**
**22281**	**CTTTACTTG**	**-**	**DEL**	**S**

**22813**	**G**	**T**	**SNP**	**S**
**23012**	**G**	**A**	**SNP**	**S**
**23063**	**A**	**T**	**SNP**	**S**
**23403**	**A**	**G**	**SNP**	**S**
**23664**	**C**	**T**	**SNP**	**S**
**23929**	**C**	**T**	**SNP**	**S**
**24095**	**G**	**T**	**SNP**	**S**
25563	G	T	SNP	ORF3a
25904	C	T	SNP	ORF3a
26456	C	T	SNP	E
27553	TTTGCACTGACTTGCTTTAGCACTCAAT TTGCTTTTGCTTGTCCTGACGGCGTAAAA CACGTCTATCAG	-	DEL	ORF7a
28253	CA	TC	SNP	ORF8
28887	C	T	SNP	N

Position	Wuhan	Delta	Type	Gene
210	G	T	SNP	ORF1ab
241	C	T	SNP	ORF1ab
1191	C	T	SNP	ORF1ab
1267	C	T	SNP	ORF1ab
3037	C	T	SNP	ORF1ab
5184	C	T	SNP	ORF1ab
9891	C	T	SNP	ORF1ab
11418	T	C	SNP	ORF1ab
12946	T	C	DEL	ORF1ab
14408	C	T	SNP	ORF1ab
15451	G	A	SNP	ORF1ab
16466	C	T	SNP	ORF1ab
18176	C	T	SNP	ORF1ab
20262	A	G	SNP	ORF1ab
**21618**	**C**	**G**	**SNP**	**S**
**21987**	**G**	**A**	**SNP**	**S**
**22029**	**AGTTCA**	**-**	**DEL**	**S**
**22917**	**T**	**G**	**SNP**	**S**
**22995**	**C**	**A**	**SNP**	**S**
**23403**	**A**	**G**	**SNP**	**S**
**23604**	**C**	**G**	**SNP**	**S**
**24410**	**G**	**A**	**SNP**	**S**
25469	C	T	SNP	ORF3a
25562	A	G	SNP	ORF3a
26767	T	C	SNP	M
27638	T	C	SNP	ORF7a
27739	C	T	SNP	ORF7a
27752	C	T	SNP	ORF7a
28248	GATTTC	-	DEL	ORF8
28271	A	-	DEL	ORF8
28461	A	G	SNP	N
28881	G	T	SNP	N

29402	G	T	SNP	N
29742	G	T	SNP	ORF10

Position	Wuhan	Omicron	Type	Gene
241	C	T	SNP	ORF1ab
2832	A	G	SNP	ORF1ab
3037	C	T	SNP	ORF1ab
3267	C	T	SNP	ORF1ab
5386	T	G	SNP	ORF1ab
6513	GTT	-	DEL	ORF1ab
8393	G	A	SNP	ORF1ab
10029	C	T	SNP	ORF1ab
10449	C	A	SNP	ORF1ab
11287	GTCTGGTTT	-	DEL	ORF1ab
11537	A	G	SNP	ORF1ab
13195	T	C	SNP	ORF1ab
14408	C	T	SNP	ORF1ab
15240	C	T	SNP	ORF1ab
18163	A	G	SNP	ORF1ab
**21762**	**C**	**T**	**SNP**	**S**
**21766**	**ACATGT**	**-**	**DEL**	**S**
**21987**	**GTGTTTATTA**	**-**	**DEL**	**S**
**22194**	**ATT**	**-**	**DEL**	**S**
**22204**	**-**	**GAGCCAGAA**	**INS**	**S**
**22578**	**G**	**A**	**SNP**	**S**
**22673**	**T**	**C**	**SNP**	**S**
**22674**	**C**	**T**	**SNP**	**S**
**22679**	**T**	**C**	**SNP**	**S**
**22813**	**G**	**T**	**SNP**	**S**
**22882**	**T**	**G**	**SNP**	**S**
**22898**	**G**	**A**	**SNP**	**S**
**22992**	**G**	**A**	**SNP**	**S**
**22995**	**C**	**A**	**SNP**	**S**
**23013**	**A**	**C**	**SNP**	**S**
**23040**	**A**	**G**	**SNP**	**S**
**23202**	**C**	**A**	**SNP**	**S**
**23403**	**A**	**G**	**SNP**	**S**
**23525**	**C**	**T**	**SNP**	**S**
**23599**	**T**	**G**	**SNP**	**S**
**23604**	**C**	**A**	**SNP**	**S**
**23854**	**C**	**A**	**SNP**	**S**
**23948**	**G**	**T**	**SNP**	**S**
**24130**	**C**	**A**	**SNP**	**S**
**24424**	**A**	**T**	**SNP**	**S**
**24469**	**T**	**A**	**SNP**	**S**
**24503**	**C**	**T**	**SNP**	**S**
**25000**	**C**	**T**	**SNP**	**S**
25584	C	T	SNP	ORF3a
26270	C	T	SNP	E
26530	A	G	SNP	M

26577	C	G	SNP	M
26709	G	A	SNP	M
27259	A	C	SNP	ORF6
28271	A	T	SNP	ORF8
28311	C	T	SNP	N
28369	AGAACGCAG	-	DEL	N
28881	G	A	SNP	N
28882	G	A	SNP	N
28883	G	C	SNP	N

Appendix Table S2 – List of primers

Name	Sequence	Target	Source
SARS-CoV-2 E gene - fwd	ACAGGTACGTTAATAGTTAATAGCGT	SARS-CoV-2 E gene	Monteil et al, 2020
SARS-CoV-2 E gene - rev	ATATTGCAGCAGTACGCACACA	SARS-CoV-2 E gene	Monteil et al, 2020
SARS-CoV-2 E gene - probe	FAM-ACACTAGCCATCCTTACTGCG CTTCG-QSY	SARS-CoV-2 E gene	Monteil et al, 2020
Human RNase P - fwd	AGATTTGGACCTGCGAGCG	Human RNase P	Monteil et al, 2020
Human RNase P - rev	GAGCGGCTGTCTCCACAAGT	Human RNase P	Monteil et al, 2020
Human RNase P-probe	FAM-TTCTGACCTGAAGGCTCT GCGCG-MGB	Human RNase P	Monteil et al, 2020

Appendix Table S3 – List of antibodies

Name	Vendor	Conjugate	Kit or product number
ACE2 capture antibody	R&D Systems	unconjugated	DuoSet ELISA Kit, Human ACE2, DY933-05
Mouse anti-His-tag	Biorad	unconjugated	MCA1396
Anti-mouse IgG	CellSignaling	HRP	#7076S
SARS-CoV-1 NP antibody (rabbit)	Sino Biological	unconjugated	# 40143-R001 – cross reactive to SARS-CoV-2
Goat anti-rabbit IgG	Life Technologies	Alexa 647	#A21245

Appendix Table S4 – p-values for Figures and Appendix Figures

Figure	Condition	p-value
**4a**	Reference 5µg/ml vs. 10µg/ml	0.0421
	Reference 5µg/ml vs. 25µg/ml	0.0081
	Reference 10µg/ml vs. 25µg/ml	0.3666
	Alpha 5µg/ml vs. 10µg/ml	0.0014
	Alpha 5µg/ml vs. 25µg/ml	0.0005
	Alpha 10µg/ml vs. 25µg/ml	0.3873
	Beta 5µg/ml vs. 10µg/ml	0.0128
	Beta 5µg/ml vs. 25µg/ml	0.0033
	Beta 10µg/ml vs. 25µg/ml	0.4126
	Delta 5µg/ml vs. 10µg/ml	0.1471
	Delta 5µg/ml vs. 25µg/ml	0.1308
	Delta 10µg/ml vs. 25µg/ml	0.9953
	Omicron 5µg/ml vs. 10µg/ml	0.1401
	Omicron 5µg/ml vs. 25µg/ml	0.0282
	Omicron 10µg/ml vs. 25µg/ml	0.4494
**4b**	Reference 5µg/ml vs. 10µg/ml	0.4903
	Reference 5µg/ml vs. 25µg/ml	0.119
	Reference 10µg/ml vs. 25µg/ml	0.5112
	Alpha 5µg/ml vs. 10µg/ml	0.9643
	Alpha 5µg/ml vs. 25µg/ml	0.0292
	Alpha 10µg/ml vs. 25µg/ml	0.0216
	Beta 5µg/ml vs. 10µg/ml	0.1182
	Beta 5µg/ml vs. 25µg/ml	0.0064
	Beta 10µg/ml vs. 25µg/ml	0.0988
	Delta 5µg/ml vs. 10µg/ml	0.2133
	Delta 5µg/ml vs. 25µg/ml	0.0962
	Delta 10µg/ml vs. 25µg/ml	0.8116
**Appendix 1a**	Ref vs. N354D	<0.0001
	Ref vs. V397F	<0.0001
	Ref vs. R408I	<0.0001
	Ref vs. W436R	<0.0001
	Ref vs. L452R	<0.0001
	Ref vs. A475V	0.4302
	Ref vs. G476S	<0.0001
	Ref vs. V483A	<0.0001
	Ref vs. F490L	0.9993
	Ref vs. N501Y	<0.0001
	Ref vs. K417N/E484/N501Y	<0.0001
**Appendix 4a**	mACE2 Wuhan	
	5 vs. 10	0.8621
	5 vs. 25	0.993
	10 vs. 25	0.9129
	mACE2 Omicron	
	5 vs. 10	0.5055
	5 vs. 25	<0.0001
	10 vs. 25	0.0011
	hACE2 Wuhan	
	5 vs. 10	0.0378
	5 vs. 25	0.0001
	10 vs. 25	0.0639

	hACE2 Omicron	
	5 vs. 10	0.3532
	5 vs. 25	0.0648
	10 vs. 25	0.6051
**Appendix 4b**	mACE2 Wuhan	
	5 vs. 10	0.2375
	5 vs. 25	0.2088
	10 vs. 25	0.9966
	mACE2 Omicron	
	5 vs. 10	0.3403
	5 vs. 25	0.0092
	10 vs. 25	0.1861
	hACE2 Wuhan	
	5 vs. 10	0.8947
	5 vs. 25	<0.0001
	10 vs. 25	<0.0001
	hACE2 Omicron	
	5 vs. 10	0.8813
	5 vs. 25	0.6869
	10 vs. 25	0.934
**Appendix 5a**	Vaccinee 1- Reference - Omicron	
	1/15	0.0006
	1/30	0.001
	1/45	0.0049
	Vaccinee 2- Reference - Omicron	0.0012
	1/135	0.0032
	1/270	0.0001
	1/540	
	Vaccinee 3- Reference - Omicron	
	1/90	0.0004
	1/180	<0.0001
	1/360	0.0001
	Vaccinee 4- Reference - Omicron	
	1/45	<0.0001
	1/90	<0.0001
	1/180	<0.0001
